# Sleep Posture and Autonomic Nervous System Activity Across Age and Sex in a Clinical Cohort: Analysis of a Nationwide Ambulatory ECG Database

**DOI:** 10.3390/s25195982

**Published:** 2025-09-26

**Authors:** Emi Yuda, Junichiro Hayano

**Affiliations:** 1 Innovation Center for Semiconductor and Digital Future (ICSDF), Mie University, Tsu 514-8507, Japan; yuda.icsdf@mie-u.ac.jp; 2 Department of Management Science and Technology, Graduate School of Engineering, Tohoku University, Sendai 982-0002, Japan; 3 Heart Beat Science Lab., Inc., Sendai 980-0845, Japan; 4 Nagoya City University, Nagoya 467-8601, Japan

**Keywords:** sleep posture, autonomic nervous system, heart rate variability (HRV), ambulatory ECG, age and sex differences, big data, wearable sensor, sleep physiology

## Abstract

**Highlights:**

**What are the main findings?**

- Sleep posture patterns varied with age and sex in a large clinical cohort of over 130,000 individuals undergoing 24-h Holter ECG monitoring.
- Posture-specific heart rate variability (HRV) indices, including HR, SDRR, HF, LF, VLF, CVHR, and Hsi, showed consistent differences between left and right lateral postures across age and sex groups.

**What is the implication of the main finding?**

- Sleep posture is a significant and independent factor influencing autonomic nervous system activity and should be considered when interpreting HRV, particularly in clinical populations.
- Incorporating posture-specific HRV analysis may enhance the physiological relevance and clinical utility of wearable ECG-based sleep monitoring.

**Abstract:**

Sleep posture has received limited attention in studies of autonomic nervous system (ANS) activity during sleep, particularly in clinical populations. We analyzed data from 130,885 individuals (56.1% female) in the Allostatic State Mapping by Ambulatory ECG Repository (ALLSTAR), a nationwide Japanese database of 24 h Holter ECG recordings obtained for clinical purposes. Sleep posture was classified as supine, right lateral, left lateral, or prone using triaxial accelerometer data. Heart rate variability (HRV) indices—including heart rate (HR), standard deviation of RR intervals (SDRR), high-frequency (HF), low-frequency (LF), very low-frequency (VLF) components, cyclic variation in heart rate (CVHR), and HF spectral power concentration index (Hsi)—were calculated for each posture and stratified by age and sex. HR was consistently lowest in the left lateral posture and highest in the right lateral posture across most age groups. Other HRV indices also showed consistent laterality, although the effect sizes were generally small. Posture distribution differed slightly by estimated sleep apnea severity, but the effect size was negligible (η^2^ = 0.0013). These findings highlight sleep posture as a statistically significant and independent factor influencing ANS activity during sleep, though the magnitude of differences should be interpreted in the context of their clinical relevance.

## 1. Introduction

Heart rate variability (HRV) analysis provides a non-invasive window into autonomic nervous system (ANS) function and has been widely applied in both physiological and clinical research. Numerous studies have demonstrated that HRV is influenced by various factors such as age, sex, circadian rhythm, sleep stages, and pathological conditions including cardiovascular and respiratory diseases [1,2,3]. Among these modulators, sleep posture has received relatively little attention, despite its known effects on cardiovascular physiology, respiratory mechanics, and sleep architecture [4,5,6].

Previous research has shown that supine posture may exacerbate obstructive sleep apnea (OSA) and that lateral postures, particularly left lateral, may improve respiratory and cardiac function during sleep [7,8,9].

However, most prior studies have been limited by small sample sizes, narrow age ranges, or laboratory-based settings, and the potential differences between the right and left lateral postures—particularly in terms of ANS activity—have not been well characterized. In the era of wearable sensors and large-scale physiological data, there is a growing opportunity to explore posture-specific variations in HRV across diverse populations and to determine whether such differences persist independently of sleep apnea status.

The Allostatic State Mapping by Ambulatory ECG Repository (ALLSTAR) is a nationwide database of over 730,000 24 h Holter ECG recordings collected for clinical purposes in Japan [10], which includes a substantial subset obtained using devices with a built-in triaxial actigraphic sensor. Leveraging this resource, we analyzed sleep posture patterns and posture-specific HRV parameters in a large, representative sample of more than 130,000 individuals spanning a wide age range.

Our objectives were threefold: (1) to characterize the distribution of sleep postures across age and sex groups, (2) to evaluate how HRV indices differ by sleep posture, and (3) to determine whether these posture-related differences are independent of potential confounders such as estimated sleep apnea severity. Understanding the influence of sleep posture on HRV may enhance the interpretation of ANS function in clinical settings and improve the utility of wearable ECG monitoring for sleep health assessment.

## 2. Materials and Methods

### 2.1. ALLSTAR Database

This study used the ALLSTAR database. The ALLSTAR project has started in 2007 in Japan and collected 738,461 Holter ambulatory ECGs recorded between November 2007 and March 2021. The 24 h ECG data in this database were recorded for some clinical purpose(s) by medical facilities and were referred for analysis to three ECG analysis centers (Suzuken Co., Ltd., Nagoya, Japan) located in Tokyo, Nagoya, and Sapporo in Japan. The data were anonymized by the centers and stored with accompanying information, including age, sex, and recording date, time, and location (postal code). Table 1 shows the characteristics of ALLSTAR subjects, including underlying cardiac diseases, cardiovascular risk factors, and medications, obtained from a randomized survey of 73,582 (10%) subjects.

The data used in this study were a subset consisting of 278,657 ECG recorded simultaneously with triaxial acceleration using wearable ECG devices with a built-in actigraphic sensor (Cardy 303 pico and Cardy 303 pico+, Suzuken Co., Ltd., Nagoya, Japan). These devices were attached to the skin on the subject’s upper chest and designed so that, when worn, their X, Y, and Z axes were always aligned with the subject’s head–foot, left–right, and front–back axes. The devices digitized three-channel ECG and triaxial acceleration at 125 and 31.25 Hz, respectively. The digitized data were analyzed with Holter ECG analyzers (Cardy Analyzer 05, Suzuken Co., Ltd., Nagoya, Japan); the temporal positions of all R waves were determined, the rhythm annotations were given to all QRS complexes, and all errors in the automated analysis were corrected manually by skilled medical technologists. The suspicious outcomes of the analysis have been reviewed by contracted cardiologists.

### 2.2. Data Selection

From the 278,657 ECG and acceleration recordings, the data for this study were selected stepwise according to the following criteria:

1. Recording duration > 5 h between 22:00 and 08:00 in lying postures (assigned as supine, right lateral, left lateral, or prone segment by the method described in Posture Estimation section below).
2. 80% of the nighttime lying data meeting criterion 1 are in sinus rhythm.

Finally, a random sample comprising 56% of the eligible data was selected. This percentage was determined based on the number of cases that could be processed within the allowable maximum computation time (one month).

### 2.3. Data Analysis

#### 2.3.1. Posture Estimation

Posture was estimated from the X-, Y-, and Z-axis components of acceleration measured at 31.25 Hz using the following procedure. First, each acceleration component was passed through a 2–3 Hz band-pass filter to extract body movement components, and the magnitude of the composite vector was calculated as the root sum of squares of the three axes. The data were divided into 30 s segments; segments in which the maximum composite vector magnitude was ≥20 mG were classified as movement segments, and the others as non-movement segments.

Next, each acceleration component was passed through a low-pass filter with a corner frequency of 0.1 Hz to extract the gravitational component. The angles between the gravity acceleration vector estimated from the three axes and each body axis were then calculated. When the angle between the head–foot axis and the gravity vector was 84° ± 36°, the posture was classified as lying. In this case, if the angle with the right–left axis was <65°, the posture was classified as left lateral; if >111°, as right lateral. If neither condition was met, the posture was classified as supine when the angle with the front–back axis was <60°, and as prone otherwise.

Finally, among the non-movement segments, those in which a single posture accounted for >50% of the segment were labeled as that posture segment; segments with no single posture exceeding 50% were classified as transition segments. The percentage of each posture for each subject was calculated as the proportion of posture-labeled segments assigned to that posture among all posture-labeled segments.

The classification performance of this model was validated using an acceleration database with known body postures measured with the same ECG device (Cardy 303 pico+, Suzuken Co., Ltd.) [11]. For supine, right lateral, left lateral, and prone postures, the model achieved recalls of 1.0, 0.90, 1.0, and 0.90, and precisions of 1.0, 1.0, 1.0, and 0.82, respectively.

#### 2.3.2. Time Domain HRV Indices

R-R interval time series were divided into 30 s segments. For each segment, heart rate (HR) and standard deviation of R-R interval were calculated using only normal-to-normal R-R intervals within the segment.

#### 2.3.3. Frequency Domain HRV Indices

The R–R interval time series, consisting only of normal-to-normal R–R intervals, was interpolated using a third-order spline function and resampled at 2 Hz. Complex demodulation [12] was then applied for three frequency bands—very low frequency (VLF; 0.003–0.04 Hz), low frequency (LF; 0.04–0.15 Hz), and high frequency (HF; 0.15–0.40 Hz)—to extract, for each band, the amplitude and instantaneous frequency as continuous functions at a resolution of 2 Hz. The continuous functions were divided into 30 s segments, and values within each segment were averaged to obtain the amplitude and instantaneous frequency of the VLF, LF, and HF components. The LF-to-HF ratio (LF/HF) was then calculated for each segment.

#### 2.3.4. Respiration Frequency Stability

The stability of respiratory frequency was estimated using the HF spectral power concentration index (Hsi) [13]. To calculate Hsi, the 2 Hz resampled R–R interval time series was divided into overlapping 256 s segments (512 points), shifted at a step of 30 s. For each segment, the power spectrum was computed using the fast Fourier transform, and Hsi was defined as the ratio of power concentrated around the spectral peak of the HF component, as described in detail in reference [13]. The Hsi value was assigned to the 30 s segment located at the center of each 256 s segment.

#### 2.3.5. Cyclic Variation in Heart Rate (CVHR)

CVHR is a characteristic pattern of HRV that accompanies sleep apnea episodes [14]. To estimate the temporal distribution of apnea-hypopnea episodes and severity of sleep apnea, CVHR was detected using a waveform analysis algorithm [15]. The hourly frequency of CVHR during total nighttime lying segments in each subject was used to estimate the apnea–hypopnea index (AHI). Subjects were classified as having normal-to-mild sleep apnea when the estimated AHI was <15/h and as having moderate-to-severe sleep apnea when the value was ≥15/h. In addition, 30 s segments were classified as sleep-apnea–positive or sleep-apnea–negative depending on the presence or absence of CVHR during the segment.

#### 2.3.6. Calculation of Indices for Each Posture

For each subject, HRV indices calculated for each 30 s segment were grouped according to the posture classification of that segment, and the mean value of each index was obtained for each posture. For CVHR, the value for each posture was calculated as the number of sleep-apnea–positive segments in that posture divided by the total hours spent in that posture.

### 2.4. Statistical Analysis

Statistical analyses were performed using the SAS Proprietary Software 9.4, SAS/STAT 12.3, SAS Institute, Cary, NC, USA). Repeated-measures ANOVA using the GLM procedure was applied to evaluate differences in preferred sleep posture by age and sex. The same procedure was also used to assess the associations between HRV and other indices with posture, as well as the effects of age and sex on these associations. Because the effect of age was not linear, age groups in 10-year intervals (AGE10) were used. In addition, to verify that the relationships between posture and HRV indices were not attributable to an association between sleep apnea and posture, differences in the proportion of each posture according to sleep apnea severity were examined using the GLM procedure, and effect sizes were evaluated using η^2^.

## 3. Results

### 3.1. Sleep Posture Distribution and Its Variation with Age and Sex

Among the 278,657 subjects, >5 h of ECG data in sinus rhythm during lying posture between 22:00 and 08:00 were available for 233,723 (84%) subjects, from which 130,885 (56%) were randomly selected for analysis. The age and sex distribution of the analyzed cohort is shown in Table 2.

**Figure 1:**
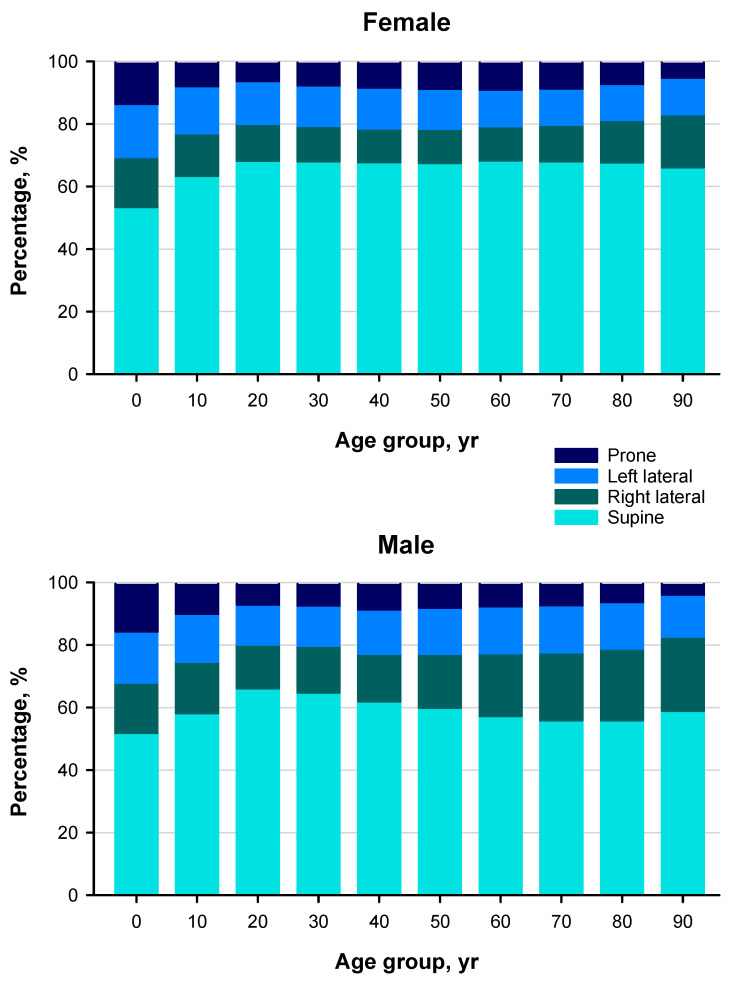
Age-related changes in sleep posture distribution by sex. Stacked bar graphs showing the percentage of time spent in each sleep posture (supine, right lateral, left lateral, prone) across 10-year age groups in females (**top**) and males (**bottom**).

In this cohort, the supine posture accounted for the largest proportion of sleep time across all age groups (Figure 1). The proportion of supine sleep was consistently higher in females than in males. The proportion of right lateral posture increased slightly with age in females after their 40s and markedly in males after their 20s. Prone posture was the least frequent in all groups and declined markedly with age in both sexes.

### 3.2. HR and SDRR Across Sleep Postures

**Figure 2:**
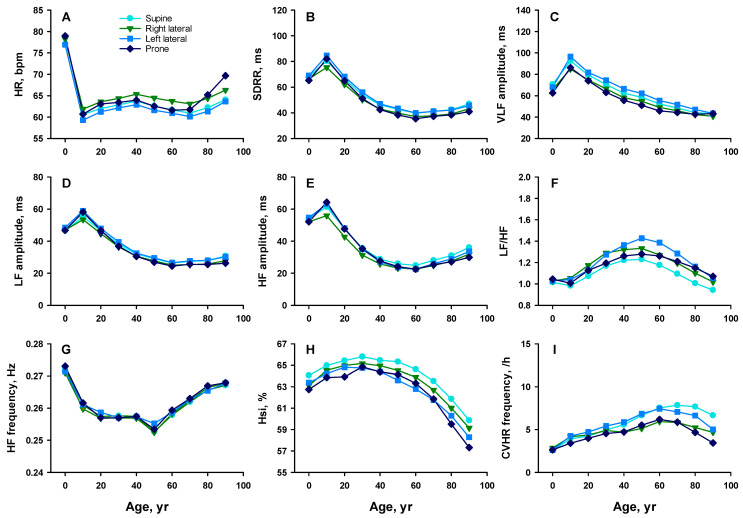
Age-related changes in HR and HRV indices by sleep posture in females. Panel (**A**): heart rate (HR); (**B**): standard deviation of R–R intervals (SDRR); (**C**): amplitude of the very low frequency (VLF) component; (**D**): amplitude of the low frequency (LF) component; (**E**): amplitude of the high frequency (HF) component; (**F**): LF-to-HF ratio (LF/HF); (**G**): frequency of the HF component; (**H**): HF spectral power concentration index (Hsi); and (**I**): frequency of cyclic variation in heart rate (CVHR). Colors indicate posture types: sky blue = supine, green = right lateral, blue = left lateral, and dark blue = prone.

**Figure 3:**
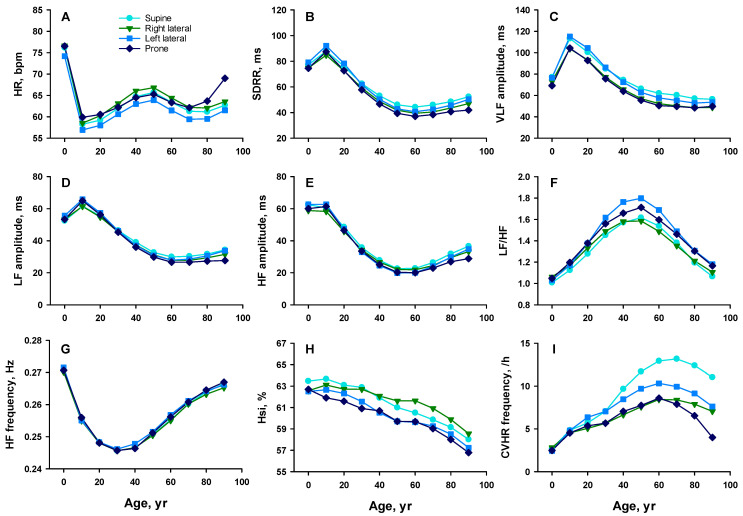
Age-related changes in HR and HRV indices by sleep posture in males. Panel (**A**): HR; (**B**): SDRR; (**C**): VLF amplitude; (**D**): LF amplitude; (**E**): HF amplitude; (**F**): LF/HF; (**G**): HF frequency; (**H**): Hsi; and (**I**): CVHR frequency. The abbreviations are described in the legend of Figure 2. Colors indicate posture types: sky blue = supine, green = right lateral, blue = left lateral, and dark blue = prone.

HR during nighttime lying varied across postures and age groups (Figure 2A and Figure 3A). In both sexes, HR was lowest in the left lateral posture across all ages and highest in the right lateral posture, except in the oldest group (≥80 years), where it was highest in the prone posture.

SDRR was highest in either the left lateral or supine posture, alternating with age, and lowest in either the prone or right lateral posture, also alternating with age (Figure 2B and Figure 3B). Between the lateral postures, SDRR was consistently higher in the left lateral posture than in the right across all age and sex groups.

### 3.3. Frequency-Domain HRV Indices Across Sleep Postures

VLF amplitude was generally highest in the left lateral posture across all age groups in females (Figure 2C and Figure 3C). In males, it was highest in the left lateral posture up to their 30s and in the supine posture thereafter. In both sexes, VLF was lowest in either the right lateral or prone posture, depending on age.

LF amplitude was highest in either the supine or left lateral posture, alternating by age, and lowest in the prone or right lateral posture in most age groups in both sexes, although the magnitude of postural differences was small (Figure 2D and Figure 3D).

HF amplitude followed a similar trend, with higher values in the supine and left lateral postures than in the right lateral and prone postures, although the magnitude of postural differences was small (Figure 2E Figure 3E).

The LF/HF varied across postures and age groups (Figure 2F and Figure 3F). In females, LF/HF was highest in the left lateral posture between their 40s and 80s, followed by the right lateral or prone posture, and lowest in the supine posture. In males, LF/HF was highest in the left lateral posture across all age groups after their 20s, followed by the prone posture, and lowest in either the supine or right lateral posture, alternating with age.

Although HF was higher in females than in males between the 10s and 40s (Figure 2G and Figure 3G), there was no significant difference with posture across all age and sex groups.

### 3.4. Hsi and CVHR Across Sleep Postures

Although Hsi was higher in females than in males between the 10s and 70s, it also exhibited posture-dependent variation (Figure 2H and Figure 3H). In females, Hsi was highest in the supine posture across all age groups. In males, Hsi was highest in the supine posture until their 30s and in the right lateral posture thereafter. In both sexes, Hsi was lowest in either the left lateral or prone posture, alternating with age.

Although the frequency of CVHR was higher in males than in females between the 20s and 70s, it also varied across postures and age (Figure 2I and Figure 3I). CVHR frequency was highest in the left lateral posture up to the 50s in females and up to the 30s in males, and was highest in the supine posture thereafter. In both sexes, CVHR frequency was lowest in either the right lateral or prone posture, alternating with age. Notably, CVHR frequency was consistently higher in the left lateral posture than in the right lateral posture.

### 3.5. Summary of Posture-Related Differences

Repeated measures ANOVA confirmed significant main effects of posture, as well as significant posture × age group interactions (*p* < 0.0001) on all HRV indices but HF (Table 3). Posture × sex interactions were generally not significant. Despite variations in absolute values with age and sex, the relative differences among postures remained consistent.

Across multiple indices—including HR, SDRR, VLF, LF, HF, LF/HF, Hsi, and CVHR—physiological differences between the right and left lateral postures were consistently observed. These included lower HR, higher SDRR, higher VLF amplitude, greater LF and HF amplitudes, higher LF/HF, lower Hsi, and higher CVHR (in adults) in the left lateral posture compared to the right. These differences persisted regardless of age and sex, and were observed in both HR and HRV indices. These results highlight laterality as a significant factor influencing cardiorespiratory ANS activity during sleep.

### 3.6. Potential Confounding by Sleep Apnea Severity

Although group differences in sleep posture proportions across estimated sleep apnea severity were statistically significant, the effect size was negligible (η^2^ < 0.0013, Table 4). Therefore, these differences are unlikely to have materially influenced the posture-dependent variations in HRV indices.

## 4. Discussion

In this large clinical cohort, sleep posture patterns varied systematically with age and sex, and posture-specific HRV indices showed consistent laterality. Across most age groups in both sexes, HR was lower and SDRR, VLF, LF, HF, and CVHR were higher in the left lateral posture compared with the right lateral posture, whereas Hsi was lower. These differences persisted after accounting for estimated sleep apnea severity, which showed negligible association with posture distribution (η^2^ = 0.0013). Although statistically robust due to the large sample size, the effect sizes were generally small, and their clinical significance should be interpreted with caution.

Our findings extend previous reports that body position influences cardiovascular and respiratory function during sleep [9,16,17]. Whereas most prior studies were limited by small samples, narrow age ranges, or laboratory settings [9], our analysis included over 130,000 individuals from a nationwide Holter ECG database with concurrent posture monitoring, enabling detailed assessment of age-, sex-, and posture-specific autonomic differences.

Notably, the relationships of SDNN, LF and HF amplitudes, and HF with age were nonlinear: these indices tended to increase until around 50–70 years of age (with the exact turning point varying by index), followed by a J-shaped trajectory. In contrast, LF/HF exhibited an inverted U-shaped (mountain-like) pattern, peaking at around 50 years of age, independent of body posture. Such nonlinear age-related changes in HRV have been reported previously [18] and are thought to be partly attributable to cardiac pacemaker instabilities, referred to as heart rate fragmentation [19]. The frequency of heart rate fragmentation increases with advancing age—particularly after 70 years—and disproportionately augments HF power, thereby distorting the apparent age trajectory of HRV indices. This mechanism may explain, at least in part, why the dimensions of nonlinearity differ among LF, HF, and LF/HF.

Several physiological mechanisms may underlie the observed laterality. In the left lateral posture, gravitational effects may enhance cardiac vagal activity through at least two mechanisms: (1) facilitating left ventricular filling, thereby increasing stroke volume and stimulating the arterial baroreceptor reflex, and (2) reducing right atrial pressure, thereby unloading the cardiac baroreceptor reflexes [20]. In addition, improved pulmonary mechanics—through reduced airway resistance and enhanced ventilation–perfusion matching [21]—could further stabilize respiration and improve oxygenation, thereby influencing HRV.

Although previous studies in cardiac patients [22,23] and elderly subjects [24] generally reported that the right lateral posture is associated with higher HF and lower LF/HF, suggesting vagal augmentation, our results showed only small HF differences and consistently lower LF/HF in the right lateral posture. Thus, our findings are only partially consistent with earlier reports and do not indicate clear vagal predominance. Several factors may account for this discrepancy, including differences in cardiac function and clinical background between study populations. In addition, posture-induced alterations in baroreceptor loading—through changes in cardiac filling pressures and venous return—could contribute. Experimental studies have demonstrated that posture can modify baroreflex bandwidth [25] and latency [26], and that asymmetric carotid loading can influence reflex cardiovascular responses [27]. Together with recent evidence questioning LF and LF/HF as direct markers of sympathetic activity [28] and suggesting that LF reflects baroreflex sensitivity [29], our findings may reflect subtle posture-related modulation of baroreflex function rather than simple sympathetic withdrawal.

The observed age- and sex-related changes in posture patterns may also be physiologically relevant. For example, the increase in right lateral posture with age in males, and the relatively stable supine preference in females, could interact with age-related changes in cardiovascular compliance, ANS control, and sleep-disordered breathing risk [22]. While supine posture is known to exacerbate OSA [9,16], lateral positioning, in general, has been shown to reduce OSA severity compared with the supine position [30,31]. However, differences in OSA severity between the left and right lateral positions have not been well characterized in previous research. Our results suggest that intrinsic autonomic differences between left and right lateral postures exist independently of OSA status.

From a clinical perspective, the present findings do not imply that posture-related HRV differences are large enough to directly guide patient management. Rather, they provide reference values indicating the extent to which variability in sleep HRV indices can be attributed to posture, stratified by age and sex. Such information may help future clinical studies disentangle posture-related variance from disease-related autonomic alterations.

As an example, sleep apnea is characterized by cyclic variation in heart rate (CVHR), the amplitude of which decreases in vagal dysfunction and has been shown to be a powerful predictor of mortality in patients with cardiovascular disease and end-stage renal disease [15]. Because the frequency of obstructive sleep apnea episodes, comorbid diseases, and aging all vary with sleep posture, it is important to consider whether the power of HRV accompanying CVHR—known to appear predominantly in the VLF band—is influenced by posture. If so, the prognostic value of CVHR amplitude may reflect not only disease severity, sleep apnea, and autonomic function but also interactions with sleep posture. Our present results provide the basis for such investigations, by clarifying the magnitude and direction of posture effects across demographic groups.

**Limitations**—This study has several limitations:

(1) Potential biases related to arrhythmia prevalence, comorbidities, and medication use may have influenced HRV indices.
(2) Sleep stages were not available, so posture-specific differences could not be disentangled from stage-specific autonomic changes (e.g., REM vs. deep sleep).
(3) Sleep apnea severity was estimated using ECG-based CVHR detection, which has lower sensitivity for mild or REM-related OSA compared with polysomnography.
(4) Posture classification was based on accelerometer thresholds, which may not capture oblique or transient positions. In addition, analysis of a dataset with known postures suggested that the method may slightly underestimate right lateral posture and overestimate prone posture.
(5) The results are not directly applicable to pulse rate variability derived from wrist-worn photoplethysmography (PPG). PRV is not a surrogate for HRV [32], partly because of variability in pulse wave velocity, which itself may be influenced by body posture and by the position of the arm wearing the device.
(6) The cohort consisted of individuals referred for clinical Holter ECG, so findings may not generalize to the healthy population.
(7) Although statistically significant, many posture-related differences had small effect sizes, and their clinical relevance should be interpreted with caution.

Future studies combining wearable ECG with validated posture and sleep stage classification, as well as simultaneous PSG validation, could help clarify these associations.

## 5. Conclusions

Sleep posture is a significant and independent determinant of HRV indices in a large clinical population. Consistent laterality in autonomic activity between left and right lateral postures was observed across age and sex groups, largely independent of sleep apnea severity. These findings suggest that posture-specific analysis should be incorporated into research protocols and clinical interpretations of ANS function during sleep, particularly as wearable ECG monitoring becomes more widespread.

Future studies should combine ECG with validated sleep staging to account for the influence of REM and deep sleep on HRV and should explore the integration of posture analysis into consumer wearable platforms. Such approaches may enhance the physiological relevance and clinical utility of posture-specific HRV assessment in both research and real-world monitoring contexts.

## Figures and Tables

**Table 1:** Characteristics of subjects in ALLSTAR database.

Cardiac Disease	Ratio, %
Coronary artery diseases	4.93
Cardiomyopathy	0.64
Valvular heart diseases	2.36
Congenital heart diseases	0.8
Heart failure	4.03
Arrhythmias	45.67
Healthy subjects (screening examination)	10.74
**Cardiovascular risk factors**	
Hypertension	37.48
Diabetes	10.29
Dyslipidemia	20.06
**Medications**	
Calcium antagonists	33.68
Angiotensin II antagonists	26.1
β blockers	8.97
Diuretics	8.46
Nitrates	6.11
Antiarrhythmic drugs	6.03
Antidiabetics	7.69
Hyperlipidemic drugs	20.92
No medication	26.66

This table was reproduced from Table 1 of a published article [10] under CC BY license. The data were obtained from a random sampling survey of 73,582 subjects (10% of the population). Subjects with multiple diseases or taking multiple medications were counted more than once.

**Table 2:** Number of subjects.

AGE10	Female	Male	Total
0	302 (48.0%)	327 (52.0%)	629 (0.5%)
10	1490 (47.0%)	1678 (53.0%)	3168 (2.4%)
20	1667 (55.4%)	1341 (44.6%)	3008 (2.3%)
30	3175 (56.3%)	2463 (43.7%)	5638 (4.3%)
40	6097 (55.4%)	4903 (44.6%)	11,000 (8.4%)
50	8024 (53.0%)	7128 (47.0%)	15,152 (11.6%)
60	14,337 (52.9%)	12,777 (47.1%)	27,114 (20.7%)
70	22,414 (57.3%)	16,728 (42.7%)	39,142 (29.9%)
80	13,941 (60.2%)	9226 (39.8%)	23,167 (17.7%)
90	1977 (69.0%)	890 (31.0%)	2867 (2.2%)
Total	73,424 (56.1%)	57,461 (43.9%)	130,885 (100%)

AGE10 = age groups in 10-year intervals.

**Table 3:** Significance of postural differences and their interactions with age and sex (repeated-measures ANOVA).

Factor	Percent of Posture	HR	SDRR	VLF Amp	LF Amp	HF Amp	LF/HF	HF Freq	His	CVHR
AGE10	-	<0.0001	<0.0001	<0.0001	<0.0001	<0.0001	<0.0001	<0.0001	<0.0001	<0.0001
Sex	-	0.2	0.3	0.002	0.09	0.2	0.0003	0.6	0.0004	0.07
Sex × AGE10	-	<0.0001	<0.0001	<0.0001	<0.0001	<0.0001	<0.0001	<0.0001	<0.0001	<0.0001
Posture	<0.0001	<0.0001	<0.0001	<0.0001	0.0007	0.01	<0.0001	0.9	<0.0001	0.001
Posture × AGE10	<0.0001	<0.0001	<0.0001	<0.0001	<0.0001	<0.0001	<0.0001	0.8	<0.0001	<0.0001
Posture × sex	<0.0001	0.1	0.7	0.4	0.7	0.7	0.5	0.9	0.4	0.2
Posture × sex × AGE10	<0.0001	<0.0001	<0.0001	<0.0001	<0.0001	<0.0001	<0.0001	0.9	<0.0001	<0.0001

AGE10 = age group divided into 10-year intervals; amp = amplitude; freq = frequency. The other abbreviations are explained in the text.

**Table 4:** Association between sleep apnea and preferred sleep posture.

Posture	Normal-to-Slight	Moderate-to-Severe	Sum of Square		Effect of Sleep Apnea	
			Effect	Total	*p*	*η* ^2^
*Female*						
Supine, %	59.1 ± 20.0	59.5 ± 20.2	303.7	10,338,480.8	0.3	0.000029
Right lateral, %	14.8 ± 14.5	13.4 ± 14.2	4168.9	5,409,296.5	<0.0001	0.000771
Left lateral, %	13.9 ± 12.6	13.5 ± 12.6	412.7	4,057,333.5	0.1	0.000102
Prone, %	12.2 ± 13.0	13.6 ± 14.2	4550.2	4,414,880.5	<0.0001	0.001031
*Male*						
Supine, %	50.5 ± 20.6	51.2 ± 20.1	1782.8	11,139,694.0	0.03	0.000160
Right lateral, %	21.4 ± 16.3	20.0 ± 15.7	8805.4	6,940,129.7	<0.0001	0.001269
Left lateral, %	16.3 ± 13.4	17.0 ± 13.6	2406.2	4,795,731.9	0.0003	0.000502
Prone, %	11.8 ± 12.8	11.8 ± 12.5	6.6	4,285,260.9	0.8	0.000002

## Data Availability

The data that support the findings of this study are available on request from the corresponding author. The data are not publicly available due to privacy or ethical restrictions of the ALLSTAR project.

## Abbreviations

The following abbreviations are used in this manuscript:

HRV	Heart rate variability
ANS	Autonomic nervous system
OSA	obstructive sleep apnea
ALLSTAR	Allostatic State Mapping by Ambulatory ECG Repository
HR	Heart rate
VLF	Very low frequency
LF	Low frequency
HF	High frequency
LF/HF	LF-to-HF ratio
Hsi	HF spectral power concentration index
CVHR	Cyclic variation in heart rate
AHI	Apnea-hypopnea index
REM	Rapid eye movement
